# The Recent Evolution of a Maternally-Inherited Endosymbiont of Ticks Led to the Emergence of the Q Fever Pathogen, *Coxiella burnetii*

**DOI:** 10.1371/journal.ppat.1004892

**Published:** 2015-05-15

**Authors:** Olivier Duron, Valérie Noël, Karen D. McCoy, Matteo Bonazzi, Karim Sidi-Boumedine, Olivier Morel, Fabrice Vavre, Lionel Zenner, Elsa Jourdain, Patrick Durand, Céline Arnathau, François Renaud, Jean-François Trape, Abel S. Biguezoton, Julie Cremaschi, Muriel Dietrich, Elsa Léger, Anaïs Appelgren, Marlène Dupraz, Elena Gómez-Díaz, Georges Diatta, Guiguigbaza-Kossigan Dayo, Hassane Adakal, Sébastien Zoungrana, Laurence Vial, Christine Chevillon

**Affiliations:** 1 Laboratoire MIVEGEC (Maladies Infectieuses et Vecteurs: Ecologie, Génétique, Evolution et Contrôle), Centre National de la Recherche Scientifique (UMR5290)—Université de Montpellier—Institut pour la Recherche et le Développement (UR 224), Montpellier, France; 2 Centre d’études d’agents Pathogènes et Biotechnologies pour la Santé (CPBS), Centre National de la Recherche Scientifique (UMR5236)—Université de Montpellier, Montpellier, France; 3 National Reference Laboratory on Q Fever, French Agency for Food, Environmental and Occupational Health Safety (ANSES), Sophia-Antipolis, France; 4 Laboratoire de Biométrie et Biologie Évolutive (LBBE), Centre National de la Recherche Scientifique (UMR5558)—Université Claude Bernard Lyon 1, Villeurbanne, France; 5 Unité d'Epidémiologie Animale, Institut National de le Recherche Agronomique (UR346), Saint Genès Champanelle, France; 6 Unité de Recherche sur les Bases Biologiques de la lutte intégrée (URBIO), Centre International de Recherche-Développement sur l'Elevage en zone Subhumide (CIRDES), Bobo-Dioulasso, Burkina Faso; 7 Biology Department, O. Wayne Rollins Research Center, Emory University, Atlanta, Georgia, United States of America; 8 Unité de Recherche sur les Maladies Infectieuses et Tropicales Emergentes (URMITE), Centre National de la Recherche Scientifique (UMR6236)—Aix Marseille Université, Dakar, Sénégal; 9 Département des Sciences et Techniques de l’Elevage (DSTE/FASE), Université Dan Dicko Dan Koulodo, Maradi, Niger; 10 Centre de Coopération Internationale en Recherche Agronomique pour le Développement (CIRAD), Prades-le-Lez, France; The Pennsylvania State University, UNITED STATES

## Abstract

Q fever is a highly infectious disease with a worldwide distribution. Its causative agent, the intracellular bacterium *Coxiella burnetii*, infects a variety of vertebrate species, including humans. Its evolutionary origin remains almost entirely unknown and uncertainty persists regarding the identity and lifestyle of its ancestors. A few tick species were recently found to harbor maternally-inherited *Coxiella*-like organisms engaged in symbiotic interactions, but their relationships to the Q fever pathogen remain unclear. Here, we extensively sampled ticks, identifying new and atypical *Coxiella* strains from 40 of 58 examined species, and used this data to infer the evolutionary processes leading to the emergence of *C*. *burnetii*. Phylogenetic analyses of multi-locus typing and whole-genome sequencing data revealed that *Coxiella*-like organisms represent an ancient and monophyletic group allied to ticks. Remarkably, all known *C*. *burnetii* strains originate within this group and are the descendants of a *Coxiella*-like progenitor hosted by ticks. Using both colony-reared and field-collected gravid females, we further establish the presence of highly efficient maternal transmission of these *Coxiella*-like organisms in four examined tick species, a pattern coherent with an endosymbiotic lifestyle. Our laboratory culture assays also showed that these *Coxiella*-like organisms were not amenable to culture in the vertebrate cell environment, suggesting different metabolic requirements compared to *C*. *burnetii*. Altogether, this corpus of data demonstrates that *C*. *burnetii* recently evolved from an inherited symbiont of ticks which succeeded in infecting vertebrate cells, likely by the acquisition of novel virulence factors.

## Author Summary

How virulent infectious diseases emerge from non-pathogenic organisms is a challenging question. Here, we address this evolutionary issue in the case of Q fever. Its causative agent, the intracellular bacterium *Coxiella burnetii*, is extremely infectious to humans and a variety of animals. However, uncertainty persists regarding its evolutionary origin, including the identity and lifestyle of its ancestors. In this article, we show that *C*. *burnetii* arose from a rare evolutionary transformation of a maternally-inherited endosymbiont of ticks into a specialized and virulent pathogen of vertebrates. While arthropod symbionts are typically transmitted maternally and thought not to be infectious to vertebrates, we establish here that one *Coxiella* symbiont has evolved the necessary adaptations to exploit the vertebrate cell, leading to the emergence of Q fever.

## Introduction

‘Query fever’ (Q fever) is a highly infectious zoonotic disease first identified in 1937 [1,2,3,4,5]. The causative agent, the obligate intracellular bacterium *Coxiella burnetii*, infects a variety of vertebrate species, including humans. Sporadic cases in humans occur annually worldwide, but occasional outbreaks are also common [1,2,3,4]. For example, in the Netherlands more than 4,000 human cases were reported between 2007 and 2010 [6]. While most human cases are self-limiting with fever and fatigue, acute forms range from mild flu-like symptoms to pneumonia or hepatitis. The disease can also become chronic (mainly endocarditis), and, though rarely fatal, remains highly debilitating even when treated with antibiotics [1,2]. Most human cases are linked to contact with infected livestock, especially goats and sheep, which suffer abortion and reproductive disorders. Infection usually occurs by the inhalation of aerosolized resistant small cell variants that are present in the excretions of infected animals. Other modes of transmission including ingestion of unpasteurized milk or dairy products; human-to-human contact is also possible but considered rare [1,2,4,5]. One of the most virulent reference strains of *C*. *burnetii* (strain RSA 493 / Nine Mile I [7]) was isolated from a guinea pig on which field-collected Rocky Mountain wood ticks *Dermacentor andersoni* had fed, suggesting that transmission through tick bites may also occur [8]. The small cell variants of the bacterium can survive and remain highly infectious for long periods in the environment, leading to the classification of *C*. *burnetii* as potential bioterrorism agent [9].

The evolutionary origin of Q fever is unclear since the *C*. *burnetii* ancestor and its primary lifestyle remain entirely unknown. Historically, *C*. *burnetii* was assigned to the taxonomic order Rickettsiales (Alphaproteobacteria), but it has been recently considered more closely related to the Legionellales order (Gammaproteobacteria) because of its genetic proximity to the Legionnaires' disease agent, *Legionella pneumophila* [10]. The Legionellales order includes many other intracellular bacteria infecting non-vertebrate species, such as, for instance, *Rickettsiella* species that are both widespread and biologically diverse in arthropods [11,12,13]. Within the *Coxiella* genus, the only known relative of *C*. *burnetii* which has been formally identified is *C*. *cheraxi*, a pathogen of crayfishes [14]. Many past descriptions of *Coxiella* were likely biased toward the detection of pathogenic strains since most *C*. *burnetii* isolates were collected from humans or domestic ruminants during Q fever outbreaks [1,5,15]. However, the advent of 16S rRNA gene sequencing as a universal DNA barcoding marker in bacteria has led to the description of a few novel *Coxiella*-like organisms in non-vertebrate species (listed in [16]), and particularly in ticks [17,18,19,20,21,22,23,24,25]. All these *Coxiella*-like organisms are closely related, but genetically distinct to *C*. *burnetii*, suggesting that some diversity exists within the *Coxiella* genus. The highly conserved nature of the 16S rRNA gene sequences has prevented researchers from establishing the exact relationship between *C*. *burnetii* and *Coxiella*-like organisms, and a sister clade relationship is commonly assumed [16,18,21,22,23].

The *Coxiella*-like organisms differ from *C*. *burnetii* in their biological traits and some may behave as subtle symbionts engaged in intricate interactions with ticks. In ticks belonging to *Ornithodoros*, *Amblyomma* and *Rhipicephalus* genera, *Coxiella*-like organisms were found to massively infect ovaries and to be maternally inherited through the egg cytoplasm [18,20,21,26]. In these tick species, the presence of the bacteria in the Malpighian tubules further suggests a possible role in nutrition by potentially provisioning their hosts with essential nutriments [20,21,26]. Indeed, the elimination of these bacteria with an antibiotic treatment was shown to negatively impact the fitness of the lone star tick *A*. *americanum* [27]. Accordingly, when the *Coxiella*-like bacterium found in *A*. *americanum* was recently sequenced [16], no recognizable virulence genes were found, indicating that this bacterium is likely non-pathogenic. In contrast, its genome encodes major vitamin and cofactor biosynthesis pathways, suggesting that it may be a vitamin-provisioning endosymbiont. This interaction exhibits the typical hallmarks of maternally-inherited symbionts with essential roles in arthropod biology [28,29]. Such patterns have been found in other exclusive blood-feeding species like bedbugs [30] and tsetse flies [31], two insect groups which rely on a single food source throughout their developmental cycle and harbor beneficial microbes that provide nutrients absent from their restricted diets. The *Coxiella*-like organisms of ticks share obvious similarities with these beneficial endosymbionts.

Here, we examine the origin of the Q fever pathogen, *C*. *burnetii*, by inferring the evolutionary processes that have shaped diversity within the entire *Coxiella* genus. To this aim, we first sampled an extensive range of ticks, with 58 tick species examined, and developed a sensitive detection method that reveals a wider *Coxiella* diversity than recognized in past studies. Second, instead of relying solely on the 16S rRNA gene, a molecular marker that is notoriously inadequate for inferring reliable fine-scale phylogenies [32], we used a novel multilocus typing method, allied to Whole Genome Sequencing (WGS) data, and conducted phylogenetic analyses on a large amount of DNA sequence data. Third, we examined two major ecological features of *Coxiella*-like organisms, i.e. their ability to be maternally-inherited through the tick egg cytoplasm and to grow in a vertebrate cell environment suitable to *C*. *burnetii*. Altogether, this corpus of data has led to the characterization of a large genetic and ecological diversity within the *Coxiella* genus, far beyond the *C*. *burnetii* type species. The *Coxiella*-like organisms of ticks form an ancient lineage of maternally-inherited tick endosymbionts that do not lie as a sister-clade to *C*. *burnetii* but rather form a basal lineage illustrative of the ancestral *Coxiella* life style.

## Results

### Ticks commonly harbor *Coxiella*-like organisms

**Fig 1:**
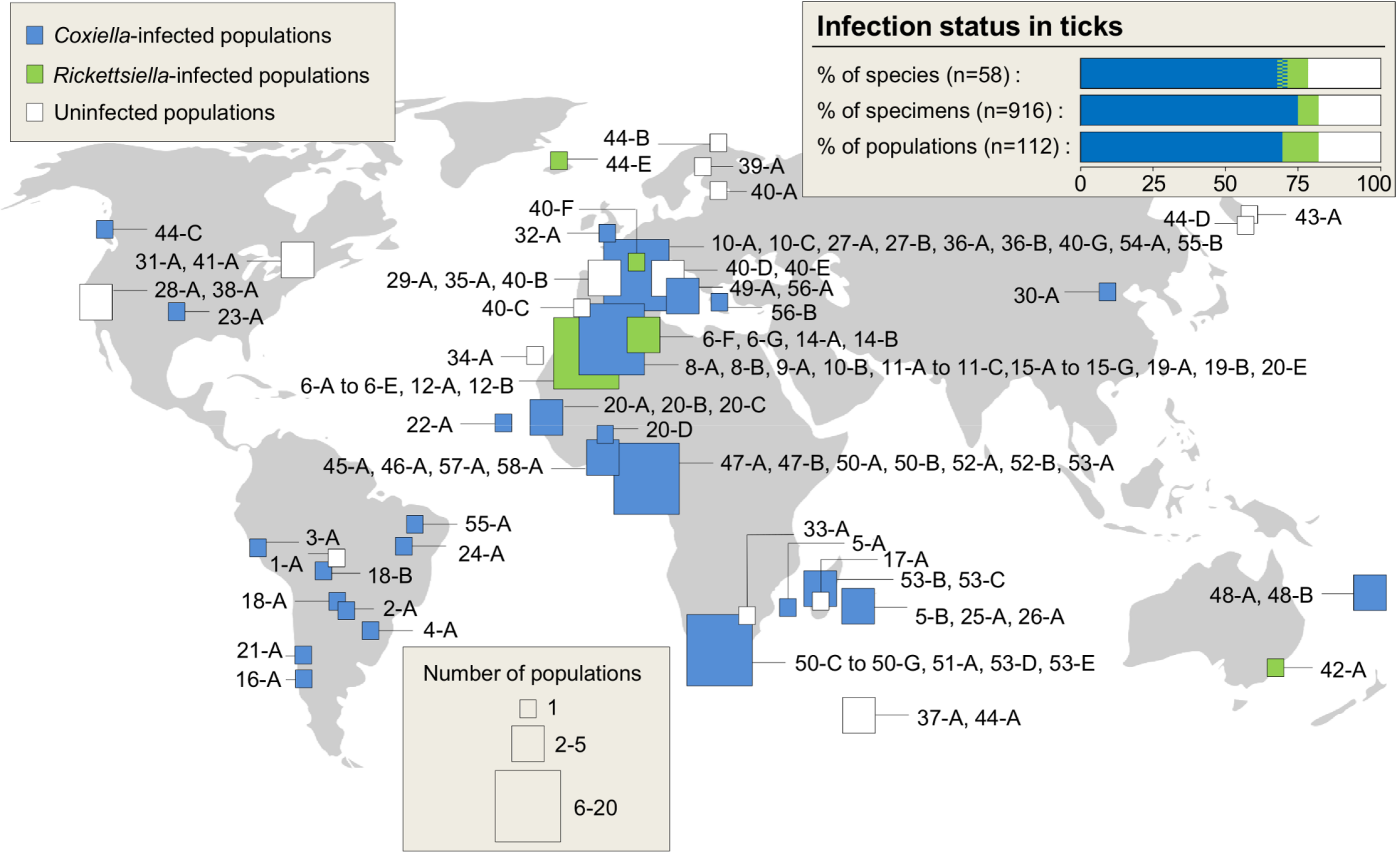
Geographic origin of the sampled ticks and distribution of *Coxiella* and *Rickettsiella* infections. Square size indicates the number of populations sampled per geographic area. Numbers refer to the tick species whereas letters discriminate the different populations screened within a species; this nomenclature is detailed in Table 1. The colors within squares indicate the infection status of populations. Two tick species were infected by both *Coxiella* and *Rickettsiella* at the species level, but not at individual and population levels.

We performed an extensive screening for the presence of *Coxiella* in 916 tick specimens from 58 species belonging to the two main tick families, Ixodidae (hard ticks, 36 species) and Argasidae (soft ticks, 22 species) (Fig 1 and Table 1). Except for 37 specimens (6 species) derived from laboratory colonies, all other tick specimens were sampled from natural populations in Europe, Americas, Africa, Oceania and Asia (n = 112 localities). In these populations, ticks were collected either in the host habitat or directly on hosts (Table 1). To detect *C*. *burnetii* and its relatives, we developed a detection method based on a nested polymerase chain reaction (PCR) using total tick DNA extracts to amplify a 539–542 base-pair (bp) fragment of the *Coxiella rpoB* gene (Table A in S1 Text).

**Table 1:** List of tick species and populations included in the analysis, with details on their origin, the population sample size, and the prevalence of *Coxiella* spp. and *Rickettsiella* spp.

Tick species		Sample sites	Tick host species or habitat	n	*Coxiella* prevalence (number of infected tick specimens)	*Rickettsiella* prevalence (number of infected tick specimens)
Argasidae (soft ticks)						
1 -	*Antricola guglielmonei* Estrada-Peña, Barros-Battesti and Venzal, 2004	1-A, Porto Velho, Rondonia, Brazil	Bat guano in cave	4	0.00 (0)	0.00 (0)
2 -	*Argas monachus* Keirans, Radovsky and Clifford, 1973	2-A, Chaco, Argentina	Unknown	3	1.00 (3)	0.00 (0)
3 -	*Ornithodoros amblus* Chamberlin, 1920	3-A, Lobos de Tierra Island, Peru, 2009	Peruvian Pelican (*Pelecanus thagus*), Peruvian Booby (*Sula variegata*)	5	1.00 (5)	0.00 (0)
4 -	*Ornithodoros brasiliensis* Aragão, 1923	4-A, Sao Francisco de Paula,Brazil	Unknown	3	1.00 (3)	0.00 (0)
5 -	*Ornithodoros capensis* Neumann, 1901	5-A, Juan de Nova Island, Mozambic Channel, 2011	Sooty Tern (*Onychoprion fuscatus*)	28	1.00 (28)	0.00 (0)
		5-B, Réunion Island	Sea bird nests	3	1.00 (3)	0.00 (0)
6 -	*Ornithodoros costalis* Diatta, Bouattour, Durand, Renaud and Trape, 2013	6-A, Kenitra, Morocco, 2006	Rodent burrows	7	0.00 (0)	1.00 (7)
		6-B, Sidi Akhfennir, Morocco, 2006	Rodent burrows	18	0.00 (0)	1.00 (18)
		6-C, Boujdour, Morocco, 2006	Rodent burrows	2	0.00 (0)	1.00 (2)
		6-D, El Argoub, Morocco, 2006	Rodent burrows	2	0.00 (0)	1.00 (2)
		6-E, Lahmiris, Morocco, 2010	Rodent burrows	1	0.00 (0)	1.00 (1)
		6-F, Mostaganem, Algeria, 2012	Rodent burrows	4	0.00 (0)	1.00 (4)
		6-G, Oudhna, Tunisia, 2010	Rodent burrows	4	0.00 (0)	1.00 (4)
7 -	*Ornithodoros denmarki* Kohls, Sonenshine and Clifford, 1965	7-H, unknown	Sea bird nests	1	1.00 (1)	0.00 (0)
8 -	*Ornithodoros erraticus* Lucas, 1849	8-A, La Calle, Tunisia, 2009	Rodent burrows	1	1.00 (1)	0.00 (0)
		8-B, Taher, Algeria, 2010	Rodent burrows	2	1.00 (2)	0.00 (0)
9 -	*Ornithodoros kairouanensis* Trape, Diatta, Bouattour, Durand and Renaud, 2013	9-A, Kairouan, Tunisia, 2010	Rodent burrows	3	1.00 (3)	0.00 (0)
10 -	*Ornithodoros maritimus* Vermeil and Marguet, 1967	10-A, Medes Island, Spain, 2009	Yellow-legged Gull (*Larus michahellis*)	20	1.00 (20)	0.00 (0)
		10-B, Zembra Island, Tunisia, 2009	Yellow-legged Gull (*Larus michahellis*)	20	1.00 (20)	0.00 (0)
		10-C, Carteau, France, 2014	Yellow-legged Gull (*Larus michahellis*)	8	1.00 (8)	0.00 (0)
11 -	*Ornithodoros marocanus* Velu, 1919	11-A, Izemmourèn, Morocco, 2009	Rodent burrows	1	1.00 (1)	0.00 (0)
		11-B, Berkane Oued Kiss, Morocco, 2006	Rodent burrows	1	1.00 (1)	0.00 (0)
		11-C, Bir-Jdid, Morocco, 2009	Rodent burrows	1	1.00 (1)	0.00 (0)
12 -	*Ornithodoros merionesi* Trape, Diatta, Belghyti, Sarih, Durand and Renaud, 2013	12-A, Guelmin, Morocco, 2006	Rodent burrows	1	0.00 (0)	1.00 (1)
		12-B, Sidi Akhfennir, Morocco, 2006	Rodent burrows	2	0.00 (0)	1.00 (2)
13 -	*Ornithodoros moubata* Murray, 1877	13-A, Laboratory strain derived from field specimens of unknown origin	Unknown	3	0.00 (0)	0.00 (0)
14 -	*Ornithodoros normandi* Larrousse, 1923	14-A, Bizerte, Tunisia, 2010	Rodent burrows	1	0.00 (0)	1.00 (1)
		14-B, Oudhna, Tunisia, 2010	Rodent burrows	2	0.00 (0)	1.00 (2)
15 -	*Ornithodoros occidentalis* Trape, Diatta, Durand and Renaud, 2013	15-A, Fes, Morocco, 2010	Rodent burrows	4	1.00 (4)	0.00 (0)
		15-B, Kenitra, Morocco, 2006	Rodent burrows	4	1.00 (4)	0.00 (0)
		15-C, Beb-Lerba, Morocco, 2010	Rodent burrows	4	1.00 (4)	0.00 (0)
		15-D, Oued Choufcherk, Morocco, 2010	Rodent burrows	4	1.00 (4)	0.00 (0)
		15-E, Bouira, Algeria, 2010	Rodent burrows	4	1.00 (4)	0.00 (0)
		15-F, Berrouaghia, Algeria, 2010	Rodent burrows	4	1.00 (4)	0.00 (0)
		15-G, Chlef, Algeria, 2010	Rodent burrows	4	1.00 (4)	0.00 (0)
16 -	*Ornithodoros peruvianus* Kohls, Clifford and Jones, 1969	16-A, Chile	Common vampire Bat (*Desmodus rotundus*)	3	1.00 (3)	0.00 (0)
17 -	*Ornithodoros porcinus* Walton, 1962	17-A, Laboratory strain derived from field specimens collected in Mahitsy, Madagascar, 2008–2010	Unknown	3	0.00 (0)	0.00 (0)
18 -	*Ornithodoros rostratus* Aragão, 1911	18-A, Salta, Argentina	Environement	18	1.00 (18)	0.00 (0)
		18-B, Laboratory strain derived from field specimens collected in Nhecolandi, Pantanal, Brazil	Environement	4	1.00 (4)	0.00 (0)
19 -	*Ornithodoros rupestris* Trape, Bitam, Renaud and Durand, 2013	19-A, Saïda Mt Daïa, Algeria, 2012	Rodent burrows	1	1.00 (1)	0.00 (0)
		19-B, Mostaganem, Algeria, 2012	Rodent burrows	2	1.00 (2)	0.00 (0)
20 -	*Ornithodoros sonrai* Sautet and Witkowski, 1943	20-A, Dielmo, Senegal, 2002	Rodent burrows	20	1.00 (20)	0.00 (0)
		20-B, Kanène Khar, Senegal, 2003	Rodent burrows	17	1.00 (17)	0.00 (0)
		20-C, Richard-Toll, Senegal, 2003	Rodent burrows	19	1.00 (19)	0.00 (0)
		20-D, Sogoli, Mali, 2007	Rodent burrows	10	1.00 (10)	0.00 (0)
		20-E, M’Chounèche, Algeria, 2009	Rodent burrows	37	1.00 (37)	0.00 (0)
21 -	*Ornithodoros spheniscus* Hoogstraal, Wassef, Hays and Keirans, 1985	21-A, Pan de Azucar, Chile, 2010–2013	Humboldt penguin (*Spheniscus humboldti*)	3	1.00 (3)	0.00 (0)
22-	*Ornithodoros* sp. (*capensis* species complex)	22-A, Boa Vista Island, Cape Verde, 2008	Cape Verde Shearwater (*Calonectris edwardsii*) and Brown Booby (*Sula leucogaster*)	16	1.00 (16)	0.00 (0)
Ixodidae (hard ticks)						
23 -	*Amblyomma americanum* (Linnaeus, 1758)	23-A, Laboratory strain derived from field engorged females collected in Oklahoma, USA, 1976–2004	Unknown	20	1.00 (20)	0.00 (0)
24 -	*Amblyomma cajennense* (Fabricius, 1787)	24-A, Chapada Gaucha, Brazil, 2013	Unknown	3	1.00 (3)	0.00 (0)
25 -	*Amblyomma loculosum* Neumann, 1907	25-A, Petite Ile, La Réunion, 2012	Wedge-Tailed Shearwater (*Puffinus pacificus*) nests	3	1.00 (3)	0.00 (0)
26 -	*Amblyomma variegatum* (Fabricius, 1794)	26-A, La Réunion	Unknown	2	1.00 (2)	0.00 (0)
27 -	*Dermacentor marginatus* (Sulzer, 1776)	27-A, Cavaillon, France, 2011	Vegetation	1	1.00 (1)	0.00 (0)
		27-B, Les Plantiers, France, 2013	Vegetation	1	1.00 (1)	0.00 (0)
28 -	*Dermacentor occidentalis* Marx, 1892	28-A, Hopland, Mendocino Co, California, 1985	Environement	6	0.00 (0)	0.00 (0)
29 -	*Dermacentor reticulatus* (Fabricius, 1794)	29-A, Proveysieux, France, 2011	Vegetation	1	0.00 (0)	0.00 (0)
30 -	*Dermacentor silvarum* Olenev, 1931	30-A, Laboratory strain derived from field specimens collected in Xiaowutai National Natural Reserve Area, China		2	1.00 (2)	0.00 (0)
31 -	*Dermacentor variabilis* (Say, 1821)	31-A, Queen's biology station, Ontario, Canada, 2013	Vegetation	2	0.00 (0)	0.00 (0)
32 -	*Haemaphysalis punctata* Canestrini and Fanzago, 1878	32-A, East Sussex, England, 2011	Vegetation	5	1.00 (5)	0.00 (0)
33 -	*Hyalomma impeltatum* Schulze and Schlottke, 1930	33-A, Zimbabwe, 1998	Zebu (*Bos indicus*)	3	0.00 (0)	0.00 (0)
34 -	*Hyalomma lusitanicum* Koch, 1844	34-A, Veneguera, Canaries, 2010	Vegetation	2	0.00 (0)	0.00 (0)
35 -	*Ixodes frontalis* (Panzer, 1798)	35-A, Bretagne, France, 2008	Eurasian Collared Dove (*Streptopelia decaocto*)	1	0.00 (0)	0.00 (0)
36-	*Ixodes hexagonus* Leach, 1815	36-A, Bretagne, France, 2008	European Red Fox (*Vulpes vulpes*)	1	1.00 (1)	0.00 (0)
		36-B, Beaumont-Monteux, France, 2008	European Hedgehog (*Erinaceus europaeus*)	2	1.00 (1)	0.00 (0)
37 -	*Ixodes kerguelenensis* André and Colas-Belcour, 1942	37-A, Crozet Archipelago, 2003	Sea bird nests	2	0.00 (0)	0.00 (0)
38 -	*Ixodes pacificus* Cooley and Kohls, 1943	38-A, Hopland, Mendocino Co, California, 1985	Environement	6	0.00 (0)	0.00 (0)
39 -	*Ixodes persulcatus* Schulze, 1930	39-A, Oulu, Finland, 2011	Vegetation	3	0.00 (0)	0.00 (0)
40 -	*Ixodes ricinus* (Linnaeus, 1758)	40-A, Lehmäsaari, Finland, 2011	Vegetation	20	0.00 (0)	0.00 (0)
		40-B, Rioja, Spain, 2011	Vegetation	20	0.00 (0)	0.00 (0)
		40-C, Mafra, Portugal, 2013	Vegetation	16	0.00 (0)	0.00 (0)
		40-D, Neuchâtel, Switzerland, 2012	Vegetation	16	0.00 (0)	0.00 (0)
		40-E, Chur, Switzerland, 2012	Hazel grouse (*Tetrastes bonasia*)	4	0.00 (0)	0.00 (0)
		40-F, Sénart, France, 2010	European Roe Deer (*Capreolus capreolus*), rodents and passerines	22	0.00 (0)	0.05 (1)
		40-G, Ain, France, 2004	Vegetation	2	1.00 (1)	0.00 (0)
41 -	*Ixodes scapularis* Say, 1821	41-A, Queen's biology station, Ontario, Canada, 2013	Vegetation	3	0.00 (0)	0.00 (0)
42 -	*Ixodes tasmani* Neumann, 1899	42-A, Kilarney Circuit, Coolangubra, Australia, 1982	Common Wombat (Vo*mbatus ursinus*)	2	0.00 (0)	1.00 (2)
43 -	*Ixodes unicavatus* Neumann, 1908	43-A, Ariy Kamen Islet, Kamchatka, Russia, 2008	Black-legged Kittiwake (*Rissa tridactyla*) and Red-legged Kittiwake (*Rissa brevirostris*)	4	0.00 (0)	0.00 (0)
44 -	*Ixodes uriae* White, 1852	44-A, Possession Island, Crozet Archipelago, 2003	King Penguin (*Aptenodytes patagonicus*)	20	0.00 (0)	0.00 (0)
		44-B, Hornoeya Island, Norway, 2010	Brünnich's Guillemot (*Uria lomvia*)	20	0.00 (0)	0.00 (0)
		44-C, Triangle Island, Canada, 2010	Rhinoceros Auklet (*Cerorhinca monocerata*)	14	0.50 (7)	0.00 (0)
		44-D, Pitchie2, Kamchatka, Russia, 2008	Red-faced Cormorant (*Phalacrocorax urile*)	20	0.00 (0)	0.00 (0)
		44-E, Grimsey, Iceland, 2003	Atlantic Puffin (*Fratercula arctica*), Common Guillemot (*Uria aalge*) and Black-legged Kittiwake (*Rissa tridactyla*)	25	0.00 (0)	0.20 (5)
45 -	*Ixodes* sp.1	45-A, Guiglo, Ivory Coast, 1994	Leopard (*Panthera pardus*)	12	1.00 (12)	0.00 (0)
46 -	*Ixodes* sp.2	46-A, Guiglo, Ivory Coast, 1994	Dog (*Canis familiaris*)	12	1.00 (12)	0.00 (0)
47 -	*Rhipicephalus annulatus* (Say, 1821)	47-A, Burkina-Faso, 2013	Zebu (*Bos indicus*)	17	1.00 (17)	0.00 (0)
		47-B, Gogonou, Benin, 2012	Zebu (*Bos indicus*)	5	1.00 (5)	0.00 (0)
48 -	*Rhipicephalus australis* Fuller, 1899	48-A, CCA Farm, New Caledonia, 2003	Cattle (*Bos taurus*)	12	1.00 (12)	0.00 (0)
		48-B, BMMMM Farm, New Caledonia, 2003	Cattle (*Bos taurus*)	12	1.00 (12)	0.00 (0)
49 -	*Rhipicephalus bursa* Canestrini and Fanzago, 1878	49-A, Italia	Cattle (*Bos taurus*) and Sheep (*Ovis aries*)	2	1.00 (2)	0.00 (0)
50-	*Rhipicephalus decoloratus* Koch, 1844	50-A, Burkina-Faso, 2013	Zebu (*Bos indicus*)	20	0.95 (19)	0.00 (0)
		50-B, Gogonou, Benin, 2012	Zebu (*Bos indicus*)	9	1.00 (9)	0.00 (0)
		50-C, Sandvelt, South Africa, 2011	Blue Wildebeest (Connochaetes taurinus), Greater Kudu (*Tragelaphus strepsiceros*) and Southern Eland (*Taurotragus oryx*)	20	1.00 (20)	0.00 (0)
		50-D, Queenstown, South Africa, 2011	Zebu (*Bos indicus*)	17	1.00 (17)	0.00 (0)
		50-E, Vaalwater, South Africa, 2010	Zebu (*Bos indicus*)	19	1.00 (19)	0.00 (0)
		50-F, Lephalale, South Africa, 2010	South African Giraffe (*Giraffa camelopardalis giraffa*)	9	1.00 (9)	0.00 (0)
		50-G, Zimbabwe, 1998	Impala (*Aepyceros melampus*)	11	1.00 (11)	0.00 (0)
51 -	*Rhipicephalus evertsi* Neumann, 1897	51-A, Zimbabwe, 1998	Zebu (*Bos indicus*)	8	1.00 (8)	0.00 (0)
52 -	*Rhipicephalus geigyi* Aeschlimann and Morel, 1965	52-A, Burkina-Faso, 2013	Zebu (*Bos indicus*)	18	0.94 (17)	0.00 (0)
		52-B, Gogonou, Benin, 2012	Zebu (*Bos indicus*)	3	1.00 (3)	0.00 (0)
53 -	*Rhipicephalus microplus* (Canestrini, 1888)	53-A, Kpinnou, Benin, 2012	Zebu (*Bos indicus*)	20	1.00 (20)	0.00 (0)
		53-B, Ambalanirana, Madagascar, 2013	Zebu (*Bos indicus*)	18	1.00 (18)	0.00 (0)
		53-C, Imeritsiatosika, Madagascar, 2013	Zebu (*Bos indicus*)	14	1.00 (14)	0.00 (0)
		53-D, Eglinton, South Africa, 2011	Zebu (*Bos indicus*)	20	1.00 (20)	0.00 (0)
		53-E, Welverdiemda, South Africa, 2011	Zebu (*Bos indicus*)	11	1.00 (11)	0.00 (0)
		53-F, Laboratory strain derived from field engorged females collected in Kpinnou, Benin, 2012	Girolando (Cattle x Zebu hybrid)	7	1.00 (7)	0.00 (0)
54 -	*Rhipicephalus pusillus* Gil Collado, 1936	54-A, Gard, France, 2006	Vegetation	2	1.00 (2)	0.00 (0)
55-	*Rhipicephalus sanguineus* (Latreille, 1806)	55-A, Brazil	Dog (*Canis familiaris*)	1	1.00 (1)	0.00 (0)
		55-B, Montferrier-sur-Lez, France, 2013	Dog (*Canis familiaris*)	1	1.00 (1)	0.00 (0)
56 -	*Rhipicephalus turanicus* Pomerantzev, 1940	56-A, Italia	Vegetation	2	1.00 (2)	0.00 (0)
		56-B, Kerkyra, Greece, 2012	Human (*Homo sapiens*)	3	1.00 (3)	0.00 (0)
57-	*Rhipicephalus* sp.1	57-A, Guiglo area, Ivory Coast, 1994	Leopard (*Panthera pardus*)	3	1.00 (3)	0.00 (0)
58-	*Rhipicephalus* sp.*2*	58-A, Guiglo, Ivory Coast, 1994	Dog (*Canis familiaris*)	2	1.00 (2)	0.00 (0)

Using this procedure, all the tick-borne bacteria we detected belong to the Legionellales order and can be unambiguously assigned either to *Coxiella* or to its sister genus, *Rickettsiella*. Whole tick DNA extracts from more than two thirds of the specimens (637 out of 916, 69.6%) and the species (40 out of 58, 70.0%) were found to be positive for *Coxiella* (Fig 1 and Table 1). *Coxiella* was found in most tested genera of hard ticks (*Rhipicephalus*, *Ixodes*, *Amblyomma*, *Dermacentor*, *Haemaphysalis*) and soft ticks (*Ornithodoros*, *Argas*). In almost all infected species, *Coxiella* was detected in >90% of the examined specimens, indicating high *Coxiella* prevalence in diverse tick species. For example, infection was apparently fixed in populations of most *Rhipicephalus* and *Ornithodoros* species (Table 1). In contrast, *Coxiella* was frequently absent in *Ixodes* species and displayed highly variable prevalence in the five infected species (out of 12 screened).

Other Legionellales bacteria of the genus *Rickettsiella* were found in 52 specimens (5.7%) from six *Ornithodoros* species and three *Ixodes* species (Fig 1 and Table 1). In two of the three *Rickettsiella*-infected *Ixodes* species, i.e. *I*. *ricinus* and *I*. *uriae*, *Coxiella* was also found, but in different individuals and in distinct populations (i.e., no co-infection by *Coxiella* and *Rickettsiella* occurred at individual and population levels; Table 1). Adding the *Rickettsiella*-positive samples, we found that 689 of the 916 examined tick specimens (75.2%) and 44 of 58 screened species (76%) harbored either *Coxiella* or one of its relatives.

### High genetic diversity among tick-borne *Coxiella*

To characterize *Coxiella* genetic diversity, we developed a multi-locus typing method based on five conserved bacterial genes including *rpoB* and four other housekeeping genes: 16S rRNA, 23S rRNA, *GroEL* and *dnaK* (Table A and Fig A in S1 Text). Multi-locus sequences were obtained from a subsample of 85 *Coxiella*- and 12 *Rickettsiella*-positive tick specimens (one to four specimens per infected species were examined). All five bacterial genes were successfully amplified from 71 *Coxiella*- and 12- *Rickettsiella* positive specimens representing 35 *Coxiella*- and six *Rickettsiella*-infected tick species. For five other *Coxiella*-infected species (i.e., 14 individual ticks), only three to four bacterial genes were successfully amplified. The sequences were easily readable without double peaks, indicating that there was no coinfection of *Coxiella*/*Rickettsiella* strains in any specimen.

The overall dataset included 33 to 40 alleles per bacterial gene (Table 2) and 51 new multi-locus genotypes (43 in *Coxiella* and eight in *Rickettsiella*). Within the *Coxiella* genus, all pairs of 16S rRNA gene sequences are at least 93% identical (Table 2) and range in threshold values typically used to delineate other Legionellales genera such as *Legionella* [33] and *Rickettsiella* [34]. Each of the infected tick species harbored a specific bacterial genotype or a set of closely related genotypes. None of the *Coxiella* multi-locus genotypes identified in ticks was identical to those of the 15 *C*. *burnetii* reference strains (Table B in S1 Text), although some showed moderate levels of nucleotide identity: pairwise identity between the two groups ranged from 77.8% to 97.7%. For each bacterial gene, the genetic diversity was significantly higher in the *Coxiella* strains of ticks than in *C*. *burnetii* as illustrated by the metrics on their respective genetic diversity (Table 2, paired t test, all P < 0.02).

**Table 2:** Genetic estimates for 85 *Coxiella*-like strains and for 15 *Coxiella burnetii* reference strains.

Locus	Function	L	Strains	N_i_	P_nsi_	N_a_	P_s_	A_d_	π	D
16S rRNA	Small ribosomal subunit	1066	*Coxiella*-like organisms	85	93.0–100	40	262	0.985	0.038	39.960
			*Coxiella burnetii*	15	99.7–100	4	4	0.552	0.001	1.224
23S rRNA	Large ribosomal subunit	496	*Coxiella*-like organisms	82	84.2–100	34	177	0.978	0.079	39.393
			*Coxiella burnetii*	15	99.4–100	2	2	0.133	0.001	0.267
*GroEL*	Chaperone protein GROEL	550	*Coxiella*-like organisms	82	68.7–100	37	292	0.982	0.179	98.527
			*Coxiella burnetii*	15	99.4–100	5	5	0.562	0.002	0.895
*rpoB*	DNA-directed RNA polymerase beta chain	474	*Coxiella*-like organisms	85	68.7–100	38	250	0.981	0.205	97.136
			*Coxiella burnetii*	15	99.3–100	4	4	0.467	0.001	0.648
*dnaK*	Chaperone protein DNAK	423	*Coxiella*-like organisms	74	69.6–100	33	227	0.979	0.177	75.789
			*Coxiella burnetii*	15	99.8–100	3	1	0.362	0.001	0.381
Full concatenated data set		3009	*Coxiella*-like organisms	71	81.8–100	36	1139	0.984	0.115	346.990
			*Coxiella burnetii*	15	99.6–100	7	16	0.781	0.001	2.933

Analyses are based on nucleotide sequences of five housekeeping genes, excluding sites with alignment gaps and/or missing data. L, sequence fragment length in base pairs; N_i_, Number of examined strains; P_nsi_, Pairwise nucleotide sequence identity (%); N_a_, number of alleles; P_s_, number of polymorphic sites; A_d_, allelic diversity; π, nucleotide diversity; D, average number of nucleotide differences between sequences.

### *Coxiella burnetii* originated from a tick-borne *Coxiella* ancestor

**Fig 2:**
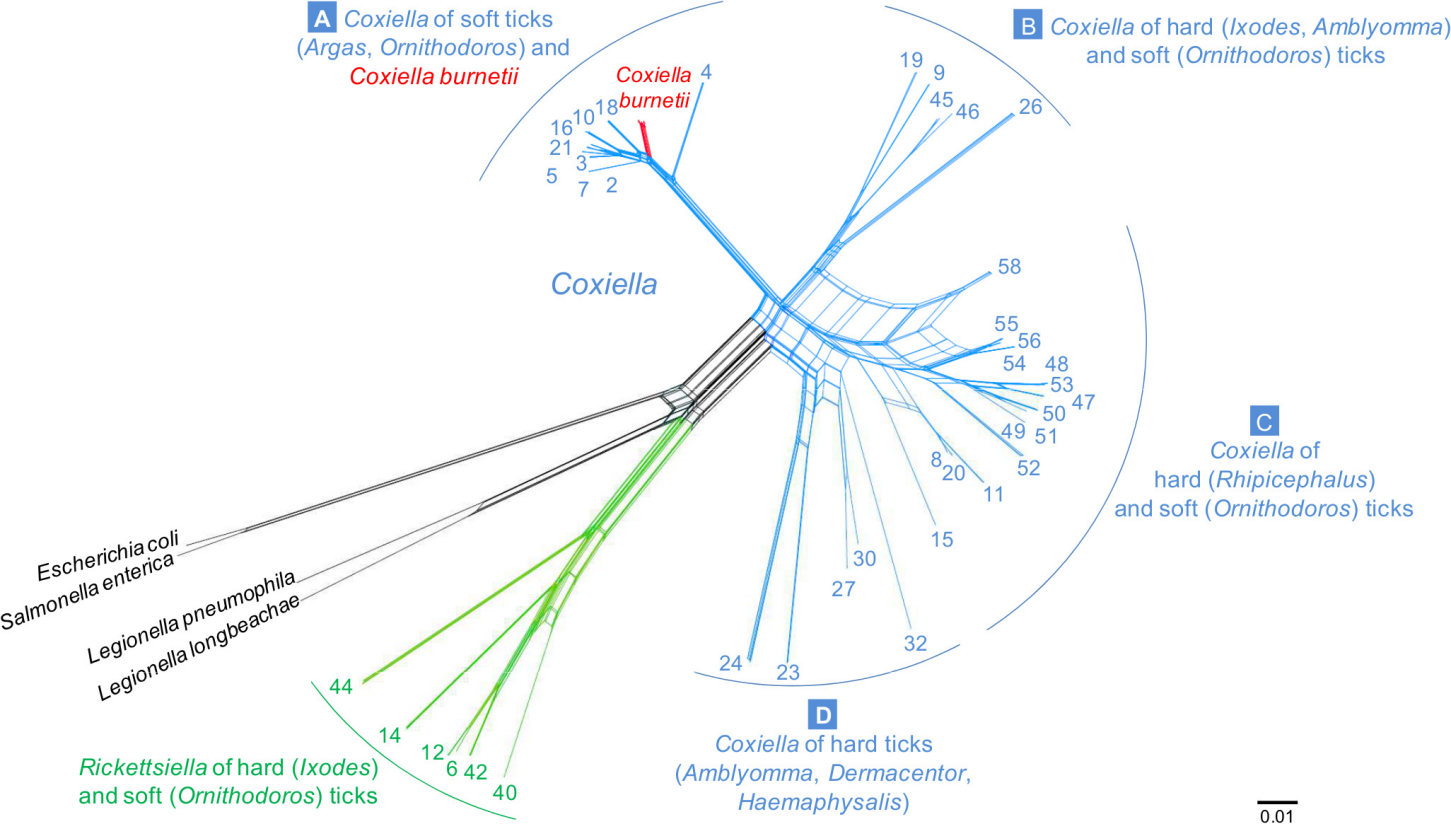
Phylogenetic network with concatenated 16S rRNA, 23S rRNA, *GroEL*, *rpoB* and *dnaK* sequences (3009 unambiguously aligned bp), including 71 *Coxiella*-like strains of ticks, 15 *C*. *burnetii* reference strains, and bacterial outgroups. The four *Coxiella* clades are labeled A to D. A zoom on the A clade which contains *C*. *burnetii* isolates is shown in Supplementary Fig C in S1 Text. Each number corresponds to one tick species as detailed in Table 1. Blue—*Coxiella*-like organisms; red—*C*. *burnetii*; green—*Rickettsiella*; black- other bacteria. All multi-locus typing of *Coxiella* and *Rickettsiella* of ticks are new sequences from this study. The scale bar is in units of substitution/site.

We constructed a multi-gene phylogeny of the entire *Coxiella* genus using a dataset that included the *Coxiella* and *Rickettsiella* sequences from ticks, the 15 *C*. *burnetii* reference genomes, as well as sequences from *Legionella* spp. and more distant outgroups that were available in GenBank (Table B in S1 Text). The concatenated sequences included 3009 unambiguously aligned base pairs (bp). Prior recombination tests showed that *Coxiella* and *Rickettsiella* strains did not exhibit a strictly clonal structure, but rather experienced significant genetic exchanges. We thus applied a sequence-based network approach that does not force relationships to be tree-like but rather incorporates recombination into the phylogenetic reconstruction. The network results (Fig 2), as well as the results from the Maximum Likelihood (ML) tree-based analysis (Fig B in S1 Text), consistently showed that the *Coxiella* genus can be split into four main clades (labeled A-to-D hereafter) with each clade clustering the *Coxiella* genotypes found in five to 15 tick species. The phylogenetic analyses also highlight that all *C*. *burnetii* isolates cluster into a unique subclade embedded within the A clade (Fig B and C in S1 Text and Fig 2). Notably, the closest relatives of *C*. *burnetii* are the *Coxiella* strains from soft ticks of the *Ornithodoros* and *Argas* genera, suggesting that the common ancestor of *C*. *burnetii* originated from a *Coxiella* hosted by soft ticks.

The partitioning of *Coxiella* diversity among tick species revealed a complex structure, indicating a role for both co-divergence and horizontal transfer events in the evolution of this bacterial group. Closely related *Coxiella*-like organisms were frequently found in closely related tick species, a pattern suggestive of co-divergence between *Coxiella* and ticks (Fig B in S1 Text and Fig 2). For instance, all the *Coxiella*-like organisms found in the 12 examined *Rhipicephalus* tick species cluster together within the C clade, whereas all the *Coxiella*-like organisms in the *Ixodes* species cluster within the B clade (Fig B in S1 Text and Fig 2). Conversely, some *Coxiella*-like organisms found in related tick species are only distantly related and do not cluster together (e.g., the *Coxiella*-like organisms of *Ornithodoros* soft ticks are scattered among the A, B and C clades), a pattern suggestive of horizontal transfers among tick species.

**Fig 3:**
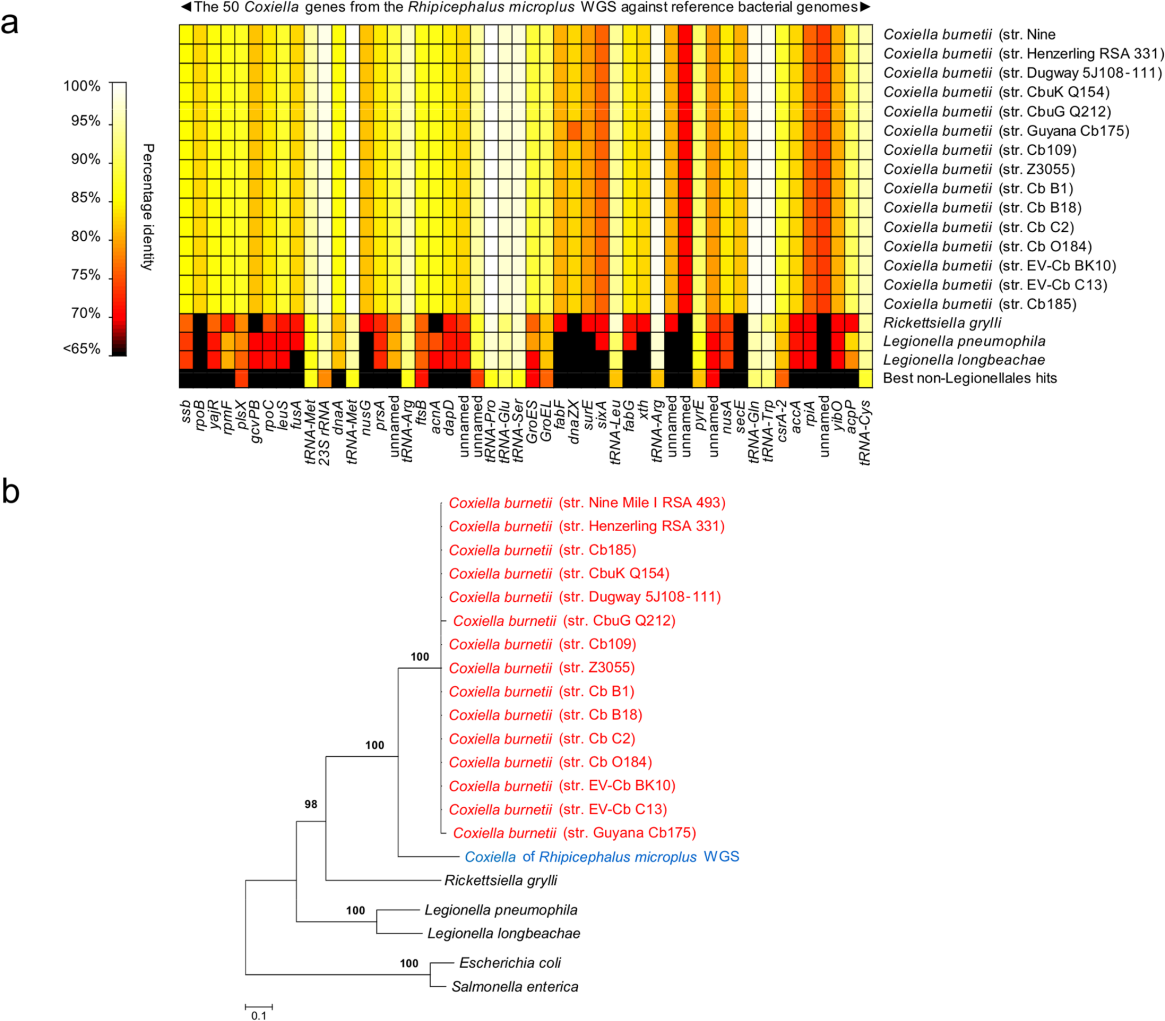
Characterization of new *Coxiella* strains derived from whole-genome sequencing (WGS) of the cattle tick *Rhipicephalus microplus*. (a) Percent identity of 50 genes uniquely attributable to *Coxiella* from *R*. *microplus* WGS versus 15 sequenced *C*. *burnetii* genomes and other reference genomes. (b) Bacterial phylogeny, comprising the *Coxiella* found in the *R*. *microplus* WGS data, reconstructed from the concatenated sequences of Fig 3A (19,304 unambiguously aligned bp) using maximum-likelihood (ML). Branch numbers indicate percent bootstrap support for major branches (1,000 replicates; only bootstrap values >90% are shown). The scale bar is in units of substitution/site.

Further analyses were conducted by examining public repositories of DNA sequencing data generated by the whole genome sequencing (WGS) projects of the cattle tick *R*. *microplus* and the deer tick *I*. *scapularis*. Using the 1,995,281 bp *C*. *burnetii* (str. Nine Mile I RSA 493) genome as a probe, we found clear evidence of *Coxiella* infections in *R*. *microplus*, but not in *I*. *scapularis*. A total of 31 contigs (514–2,349 bp, totaling 34,990 bp) from *R*. *microplus* sequencing were uniquely attributable to *Coxiella*. They matched 50 genes of *C*. *burnetii* with 68-to-100% nucleotide identity (Fig A and Table C in S1 Text and Fig 3A). Alignment of the 31 *Coxiella* contigs to other bacterial genomes, including the 15 *C*. *burnetii* reference genomes (19,304 unambiguously aligned bp), corroborates the finding of our prior five loci-based analyses: the *Coxiella* strain identified in *R*. *microplus* is evolutionarily related, but distinct, to *C*. *burnetii* (Fig 3B).

### *Coxiella*-like organisms are maternally inherited in ticks

It should be noted that the *R*. *microplus* WGS DNA examined above was extracted from eggs of an inbred strain of ticks (Deutsch strain), first derived from a few field specimens sampled in Texas in 2001, and reared for at least seven generations in the laboratory. The presence of *Coxiella* DNA in the WGS of *R*. *microplus* eggs thus raised the issue of their maternal inheritance in ticks. To address this question, 24 gravid females of four *Coxiella*-positive tick species were collected either from seabird nests (*O*. *maritimus*, n = 8 females), from a dog (*R*. *sanguineus*, n = 1) or from laboratory colonies (*R*. *microplus*, n = 7; *A*. *americanum*, n = 8) in order to test for the presence of *Coxiella*-infection in the cytoplasm of their progeny (8 to 14 surface-sterilized eggs per female were individually examined; i.e., 244 eggs in total). The occurrence of maternal transmission was detected in all four tick species and in almost all eggs: *O*. *maritimus*—79 *Coxiella*-positive eggs out of 80, *R*. *sanguineus*—14 out of 14, *R*. *microplus*—68 out of 70, and *A*. *americanum*—80 of 80. The mean transmission rate can thus be estimated at 0.988 (95% confidence interval, 0.965–0.994), demonstrating highly efficient maternal transmission of *Coxiella* in ticks. Maternal inheritance is thus widespread in the *Coxiella* genera, being found in three different clades (A: *Coxiella*-like organism of *O*. *maritimus*; C: *R*. *sanguineus* and *R*. *microplus*; D: *A*. *americanum*).

### Differences in metabolic requirements of *Coxiella*-like organisms and *C*. *burnetii*

We next compared the metabolic requirements of *Coxiella*-like organisms with those of *C*. *burnetii* by assessing their ability to replicate in both an axenic medium ACCM2 (mimicking the environment of the acidified lysosome-like vacuoles of phagocytes typically colonized by *C*. *burnetii*; [35]) and directly inside vertebrate host cells. First, ACCM2 was inoculated with *Coxiella*-like organisms extracted from eggs of either *O*. *maritimus*, *R*. *microplus* or *A*. *americanum*. Inoculated media were incubated for 10 days under standard conditions used to amplify *C*. *burnetii*. Although our *C*. *burnetii* positive controls readily replicated in the media, the *Coxiella* obtained from eggs of the three tick species did not grow. We then incubated egg homogenates from ticks of *O*. *maritimus* and *R*. *microplus* with mammalian cell cultures for seven days. Similar to results under axenic conditions, the incubation of vertebrate cell lines with *Coxiella*-like organisms failed to produce *Coxiella*-containing vacuoles, whereas the same cell lines incubated with *C*. *burnetii* under the same conditions were readily infected. The apparent inability to amplify tick-borne *Coxiella* through standardized protocols, well-characterized for *C*. *burnetii*, suggests that, despite their phylogenetic proximity, the *Coxiella*-like bacteria are adapted to radically different environments.

## Discussion

Since its original description, *C*. *burnetii* infections have been characterized in a wide variety of hosts. While only two species have been formally identified within the *Coxiella* genus, we show here that a far greater diversity of *Coxiella* exists in ticks. We detect the presence of *Coxiella*-like organisms in many more tick species than previously known [17,18,19,20,21,22,23,24,25] and describe a far wider genetic diversity among these bacteria than previously suspected. The incidence of *Coxiella*, as well as of its sister genus *Rickettsiella*, in ticks is exceptionally high, with approximately three quarters of tick species infected. Although possible tick-borne transmission of *C*. *burnetii* has been reported [1,2,8], none of the 43 new *Coxiella* genotypes identified here are identical to *C*. *burnetii*. We also demonstrate for genetically divergent *Coxiella* strains (i.e., members of the A, C and D clades) found in four tick species that infection is primarily transmitted maternally via the egg cytoplasm. These results converge to support the hypothesis that these *Coxiella*-like organisms are specific endosymbionts of ticks. Phylogenetic evidence further shows that one of the *Coxiella*-like organisms belonging to the A clade and primarily hosted by soft ticks has served as the progenitor of *C*. *burnetii*.

Three complementary lines of argument indicate a much longer evolutionary history for *Coxiella*-tick associations than for vertebrate-*Coxiella* associations. The first lies in the broad distribution of *Coxiella* and *Rickettsiella* bacteria across tick species, genera and families. The second concerns the extensive genetic diversity found in tick-borne *Coxiella* strains compared to *C*. *burnetii* strains, as illustrated by the clear subdivision of this genus into four highly divergent clades (A-D). Finally, the clustering of all *C*. *burnetii* strains within one of the clades of tick-borne *Coxiella* shows that the ancestor of *C*. *burnetii* was a tick-associated bacterium which succeeded in infecting vertebrate cells. The remarkably low genetic diversity of *C*. *burnetii*, previously noted in other studies [36,37], indicates a unique and recent emergence of this highly infectious vertebrate pathogen. Interestingly, this hypothesis was initially raised a decade ago from observations of the profound differences in genome architecture of *C*. *burnetii* relative to other pathogenic intracellular bacteria [38]. It was again emphasized from the genome sequencing of new *C*. *burnetii* strains [39]. Our data brings further support to this hypothesis by demonstrating that *C*. *burnetii* roots within the *Coxiella* phylogeny. Comparative genome sequences of *C*. *burnetii* [38, 39] and of the *Coxiella*-like organism from *A*. *americanum* [16] also suggest that *Coxiella* bacteria differ substantially in terms of genome size and gene content. The *C*. *burnetii* genome (A clade) has a size of ca 2Mb [38, 39], whereas the genome of the *Coxiella*-like organism isolated in *A*. *americanum* (D clade) is only about a 1/3 of this size (ie. 0.66 Mb) with a large percentage of missing genes [16]. This reduction in genome size may limit the transition to pathogenicity, and suggests that some *Coxiella*-like organisms may have evolved towards exclusive and irreversibly specialized interactions with their tick hosts. Overall, the diversity of genome sizes emphasizes that members of the different *Coxiella* clades may have retained a variety of evolutionary strategies to favour their spread and persistence in their hosts.

We identified *Coxiella* as a major emerging clade of bacterial endosymbionts allied to ticks. *Coxiella*-like organisms are maternally-transmitted through the egg cytoplasm at high frequency with 98–100% mother-to-offspring transmission, a pattern also reported in previous studies [20,21,26]. This transmission pattern is the rule for a variety of bacterial endosymbionts that live exclusively within arthropod cells [28,29,40]. While some, like *Wolbachia*, are globally common symbionts estimated to infect ca. 40% of insect species [41,42], others are globally rare, but common and important in particular arthropod groups [28]. This is precisely the case for *Coxiella*-like organisms; although they have not been found in other arthropod species, they are commonly associated with ticks. This leads to the obvious question of the phenotypic consequences of *Coxiella*-tick interactions. In some cases, *Coxiella*-like organisms of ticks likely act as obligate mutualistic symbionts required to support normal tick development, potentially provisioning their hosts with essential nutriments absent in vertebrate blood [16,20,21,26,27]. The ubiquity of *Coxiella* in some tick groups-such as in the *Rhipicephalus* genus in which infection is at fixation- corroborates the hypothesis of an obligate endosymbiont. This is not, however, the case for all tick species since some, such as *I*. *ricinus* and *I*. *uriae*, harbour *Coxiella*-like organisms at much lower frequencies. In these tick species, *Coxiella* is more likely to behave as a conditional mutualist-i.e., that confers advantages under certain environmental conditions- or as a reproductive parasite-i.e., that manipulates host reproduction toward the production of daughters (the transmitting sex), as commonly observed in arthropods with a variety of facultative symbionts [28,40]. It should also be noted that other endosymbionts also occur in ticks and may have evolved under complex multispecific interactions [17,24]. For instance, whereas the soft tick *O*. *moubata* was not found to be infected by a *Coxiella*-like organism in the present study, this tick species has been found to be infected by an endosymbiont belonging to the *Francisella* genus [17]. Endosymbionts other than *Coxiella* may thus interact with ticks, a pattern suggesting that endosymbiotic systems can be dynamic across tick lineages. These different hypotheses will now require specific testing.

Another question remains concerning the degree of vertebrate infection risk by the *Coxiella*-like organisms of ticks. Ticks are found worldwide and blood-feed on many different hosts; a combination of traits that may facilitate tick-to-vertebrate transfers of *Coxiella*. However, the bacteria observed in this study seem confined to ticks and, to our knowledge, none have ever been isolated from a vertebrate or associated with clinical symptoms. This suggests that these tick-associated bacteria currently pose a much lower infection risk to vertebrates than *C*. *burnetii*. As discussed above, the genome reduction of the *Coxiella*-like organism isolated in *A*. *americanum*, with the lack of nearly all the genes associated with pathogenicity [16], corroborates this view. Moreover, the inability to grow tick-borne bacteria in vertebrate cells highlights the significant barrier that must be overcome by the bacteria to successively achieve tick-to-vertebrate transmission. This type of transmission may, nonetheless, occasionally occur; an avian *Coxiella*-like organism was recently reported to induce fatal systematic infections in domestic birds [43,44,45]. A very similar infection pattern was found for another maternally inherited endosymbiont, *Arsenophonus*, a widespread bacterium in different insect groups [41,46,47,48,49]. In particular, some *Arsenophonus* strains were detected in the phloem of plants fed on by infected phytophagous insects and were assumed to be opportunistic plant pathogens [50]. In such cases, the plant host may act as an ecological arenas for the global exchange of endosymbionts like *Arsenophonus*, serving as a possible intermediate host for the horizontal transfer of bacteria among insect species [48]. In the case of *Coxiella*-like organisms, the extent of exchange between different tick species via the vertebrate host is yet to be established, but could be favoured by tick co-feeding (ticks feeding in close proximity on the host). The genetic similarity between *Coxiella*-like organisms found in unrelated tick species highlights the capacity to shift tick host species. Future research is now needed to assess the potential of different *Coxiella*-like organisms to infect vertebrates.

The reasons why *C*. *burnetii* is a highly virulent pathogen of vertebrates, but not *Coxiella*-like organisms (especially those from the A clade) remain unknown. As an intracellular pathogen with airborne transmission, *C*. *burnetii* has evolved specific mechanisms to survive in the abiotic environment, as well as to infect and exploit vertebrate cells [15]. Several evolutionary pathways may explain the acquisition of the genetic material necessary for this major lifestyle transition; this includes spontaneous genetic mutations in the genome of a tick-*Coxiella* ancestor, or the more likely transfer and integration of virulence genes from a co-infecting pathogen. The opportunity of gene transfer among bacteria, irrespective of their pathogenic or symbiotic properties, relies on their frequent co-occurrence within the same tick host [25,51,52]. The *Coxiella*-like organisms of the A clade may have dynamic genomes as observed in many arthropod symbionts: although they reside in confined intracellular environments, arthropod symbionts commonly experience variable degrees of recombination and gene transfer [53,54,55,56,57]. These gene transfers have served as immediate and powerful mechanisms of rapid adaptation in many endosymbionts, such as *Wolbachia* [56] and *Hamiltonella* [55,57]. This mechanism may explain the evolutionary transition from a *Coxiella* tick-symbiont of the A clade to the vertebrate pathogen *C*. *burnetii*. Other genetic connections are also possible; several *C*. *burnetii* genes that may contribute to major virulence traits, such as tissue tropism, are similar to eukaryotic genes and may have been acquired through lateral gene transfers from eukaryotes [38,39]. Detailed studies of virulence genes in *C*. *burnetii* and their homology with *Coxiella*-like organisms of the A clade will now be necessary to understand the remarkable emergence of the Q fever agent.

The evolutionary transition observed within the *Coxiella* genus is one of the rare cases reported to date of an arthropod-inherited symbiont evolving metabolic adaptations leading to the emergence of a vertebrate infectious disease. Another such transition occurred in the *Rickettsia* genus. The best-known members of this genus are transmitted by blood-feeding arthropods and are pathogenic in the vertebrate host. However, in recent years, many maternally-inherited *Rickettsia* endosymbionts found exclusively in arthropods have been discovered [58,59]. The examination of the evolutionary history of the *Rickettsia* genus revealed that this bacterium originated from endosymbionts of invertebrates and only secondarily became vertebrate pathogens [58,59]. Like *Coxiella*, some *Rickettsia* species of blood-feeding hosts have underwent a horizontal transmission through a vertebrate host, leading to pathogen emergence. Other bacteria, such as *Arsenophonus* [47,48] and *Sodalis* [60] may have had similar life cycle transitions, but the case of *Coxiella* is unique in that the arthropod host is no longer required to complete its life cycle.

In conclusion, we show that *C*. *burnetii* arose from a rare and recent event: the evolutionary transformation of a maternally inherited endosymbiont of ticks into a specialized and virulent pathogen of vertebrates. This raises a series of exciting questions related to both how *Coxiella* endosymbionts made the major evolutionary transition leading to the emergence of Q fever and their role in the population dynamics of ticks. Identifying the evolutionary processes that transform symbiotic bacteria into emerging pathogens will require further exploration into the biology of the entire *Coxiella* genus.

## Methods

### Tick collection

The examined specimens represent the two main tick families, nine genera, 58 species and 112 populations from around the world (Table 1). Field specimens were sampled on various host species belonging to major mammal and bird families or from their habitats. We also used specimens from laboratory colonies reared in captivity for at least three generations for six tick species (derived from field specimens collected in North America, South America, Africa and China). All samples were preserved in 70–90% ethanol at room temperature until use. Before storage, tick eggs collected under laboratory conditions were surface-sterilized with 2.6% sodium hypochlorite and 0.5% SDS for 1 min and washed with sterile water to avoid external bacterial contamination.

### *Coxiella* screening and typing

Tick DNA was individually extracted using the DNeasy Blood & Tissue Kit (QIAGEN) following manufacturer instructions. DNA template quality was systematically verified by PCR amplification of the 18S ribosomal RNA (18S rRNA) or the cytochrome oxydase 1 (*C01*) arthropod primers (Table A in S1 Text). Tick DNA samples were then tested for *Coxiella* presence using a nested PCR assay and sequencing of the *rpoB* gene using *Coxiella*-specific primers. The use of nested PCR was efficient at decreasing the probability of contamination from unwanted amplification products. Additional PCR assays on the 16S rRNA, 23S rRNA, *GroEL* and *dnaK* genes were conducted on a subsample of *Coxiella*-positive tick DNA to obtain additional DNA sequences for phylogenetic analyses. We used 15 recently published genomes of *C*. *burnetii* (mainly isolated from humans and ruminants) and the genome of *Rickettsiella grylli* from woodlice (listed in Table B in S1 Text) as references to design PCR primers. The efficiency of our typing method was ascertained through positive PCR amplification and clear sequences for the five loci in four cultured reference strains of *C*. *burnetii* (Table B in S1 Text). Gene features, primers and PCR conditions are detailed in Table A in S1 Text. All PCR products were visualized through electrophoresis in a 1.5% agarose gel. Positive PCR products were purified and sequenced in both directions (EUROFINS). The chromatograms were manually inspected and cleaned with CHROMAS LITE (http://www.technelysium.com.au/chromas_lite.html) and sequence alignments were done using CLUSTALW [61], both implemented in MEGA [62].

*Coxiella* sequences were also searched for in the whole genome sequence (WGS) data of *R*. *microplus* and *I*. *scapularis* (GenBank accession numbers ADMZ02000000 and ABJB000000000, respectively) using the 1,995,281 bp *C*. *burnetii* genome (str. Nine Mile I RSA 493, GenBank accession number NC002971) as a probe and the Basic Local Alignment Search Tool (BLAST) with default parameters. Table C in S1 Text reports the number and content of *Coxiella* contigs that were detected in the *R*. *microplus* WGS data.

### Molecular and phylogenetic analyses

The GBLOCKS program [63] with default parameters was used to remove poorly aligned positions and to obtain non-ambiguous sequence alignments. Sequences of individual genes that differed by one or more nucleotides were assigned distinct allele numbers using DNASP [64], with the option of excluding sites with alignment gaps and/or missing data. Tick-borne *Coxiella* strains are defined as each unique combination of alleles. The genetic diversity estimates (P_s_, number of polymorphic sites; A_d_, allelic diversity; π, nucleotide diversity; D, average number of nucleotide differences between sequences) were computed using DNASP. Other statistical analyses were carried out using the R statistical package. All sequence alignments were checked for putative recombinant regions using the GENECONV [65] and RDP [66] methods available in the RDP3 computer analysis package [67].

Phylogenetic analyses were based on single and concatenated sequences of the five bacterial genes used in the multi-locus typing scheme and on the 50 *Coxiella* genes found in the *R*. *microplus* WGS data. Sequence alignments included *Coxiella* and *Rickettsiella* sequences obtained in this study from tick DNA, as well as sequences available in GenBank from reference strains of *C*. *burnetii*, *Rickettsiella grylli*, *Legionella pneumophila*, *L*. *longbeacheae*, and two more distantly related bacteria, *Escherichia coli* and *Salmonella enterica* (Table B in S1 Text). The evolutionary models fitting the sequence data most closely were determined using the Akaike information criterion with the program MEGA. For each data set examined, the best-fit approximation was the general time reversible model with gamma distribution and invariant sites (GTR+G+I). Network-based phylogenetic analyses were done using SplitsTree, implementing the evolutionary model under the agglomerating NeighborNet algorithm [68]. Tree-based phylogenetic analyses were done using maximum-likelihood (ML) analyses. A ML heuristic search using a starting tree obtained by neighbor-joining was conducted in MEGA. Clade robustness was assessed by bootstrap analysis using 1,000 replicates.

### Culture assays

We first assessed the ability of tick-borne *Coxiella* to replicate in an axenic medium as follows. Tick eggs were surface-sterilized as described above and homogenized by hand in sterile water. Eggs homogenates were used to inoculate 2ml of the axenic medium ACCM2 [35] and incubated three days in a humidified atmosphere of 5% CO2 and 2.5% O2 at 37°C. 50μl of each culture were then diluted in 2ml of fresh ACCM2 and further incubated under the same conditions for 10 days to assess bacterial growth. We then assessed the ability of tick-borne *Coxiella* to replicate inside vertebrate host cells as follows. Surface-sterilized tick eggs were homogenized by hand in 1 ml of 10% Fœtal Bovine Sérum (FBS) supplemented MEM medium (GIBCO). The homogenate (0.5 ml) was diluted in 25 ml of 10% SVF-MEM and centrifuged at 4000 rpm (2000g) at 4°C for 30 min. Ten ml of the supernatant was mixed with 10 ml of 10% FSB-MEM and again centrifuged at 2000g at 4°C for 30 min. Ten ml of the supernatant was harvested and filtered through a sterile 0.45 μm pore size filter (MILLIPORE). Two flasks containing confluent Sheep Fœtal Thymus cells (SFT) cells were inoculated with 5 ml of the obtained filtrate and incubated at 35°C and allowed to grow for 12 weeks. Cell culture flasks were observed daily for the presence of contamination or growth signs such as vacuoles containing *Coxiella*, during the first week then once a week. As a positive control, a homogenate of *C*. *burnetii* was used following the same protocol.

### Accession codes

Nucleotide sequences of PCR-amplified fragments of tick-borne *Coxiella* and *Rickettsiella* genes have been deposited in the GenBank nucleotide database under accession codes KP994768-KP994862 (16S rRNA), KP994678-KP994767 (23S rRNA), KP985445-KP985537 (*GroEL*), KP985265-KP985357 (*rpoB*) and KP985358-KP985444 (*dnaK*).

## Supporting Information

S1 Text Table A. Genes and primers used in polymerase chain reaction (PCR) assays to detect Coxiella and relatives and to control tick DNA quality. The same primers were used for the *Coxiella* and *Rickettsiella* typing, with the exception of the 16S rRNA gene for which different primers were designed for the two bacteria. Nested PCR amplifications (16S rRNA, 23S rRNA, *GroEL*, *rpoB* and *dnaK*) were performed as follows: the first PCR run with the external primers was performed in a 10μLvolume containing 20–50 ng of genomic DNA, 3 mM of each dNTP (Thermo Scientific), 8 mM of MgCl_2_ (Roche Diagnostics), 3 μM of each primer, 1 μL of 10× PCR buffer (Roche Diagnostics), and 0.5 U of Taq DNA polymerase (Roche Diagnostics). A 1-μL aliquot of the PCR product from the first reaction was then used as a template for the second round of amplification. The second PCR was performed in a total volume of 25 μL and contained 8 mM of each dNTP (Thermo Scientific), 10 mM of MgCl_2_ (ThermoScientific), 7.5 μM of each of the internal primers, 2.5 μL of 10×PCR buffer (Thermo Scientific), and 1.25 U of Taq DNA polymerase (Thermo Scientific). Non-nested PCR amplifications (CO1 and *18S rRNA*) were performed following conditions similar to the first PCR run used in the nested PCR assays. All PCR amplifications were performed under the following conditions: initial denaturation at 93°C for 3 min, 35 cycles of denaturation (93°C, 30 s), annealing (Tm = 50–56°C, depending on primers, 30 s), extension (72°C, 1–2 min), and a final extension at 72°C for 5 min. **Table B.** List, biological features and GenBank accession numbers of the bacterial strains used as references in molecular and phylogenetic analyses.* reference strains of *C*. *burnetii* used for primer testing. **Table C.** List, sequence accession numbers and features of the 31 *Coxiella* contigs from the whole-genome shotgun sequencing (WGS) of the cattle tick *Rhipicephalus microplus*. **Fig A.** Map of the *Coxiella burnetii* genome (strain Nine Mile I RSA 493) showing the position of the genetic markers (in blue) used in this study. The arrows indicate the position along the chromosome of the five housekeeping genes (16S rRNA, 23S rRNA, *GroEL*, *rpoB* and *dnaK*) used in the multi-locus typing of tick-borne *Coxiella* infections. The numbered boxes (1–31) indicate the position of the 31 *Coxiella* contigs (listed in Table C in S1 Text) detected from the whole genome sequencing of the hard tick *Rhipicephalus microplus*. **Fig B.** *Coxiella* and *Rickettsiella* phylogeny constructed using maximum-likelihood (ML) estimations based on 16S rRNA, 23S rRNA, *GroEL*, *rpoB* and *dnaK* concatenated sequences (3009bp), including 71 Coxiella-like strains of ticks, 15 *C*. *burnetii* reference strains and outgroups. The four *Coxiella* clades are labeled A to D. Each number corresponds to one tick species as detailed in Table 1. Blue, *Coxiella*-line organisms; red, *C*. *burnetii*; green, *Rickettsiella*; black, other bacteria. All multi-locus typing of tick-borne *Coxiella* and *Rickettsiella* of ticks are new sequences from this study. Branch numbers indicate percentage bootstrap support for major branches (1000 replicates; only bootstrap values >90% are shown). **Fig C.** Inset of *Coxiella* network from Fig 2 with focus on the A clade (*Coxiella* of soft ticks and *C*. *burnetii*). Each number corresponds to one tick species as detailed in Table 1. Blue, *Coxiella*-line organisms; red, *C*. *burnetii*. The scale bar is in units of substitution/site.
(DOCX)
