# Supplementary material for: The Recent Evolution of a Maternally-Inherited Endosymbiont of Ticks Led to the Emergence of the Q Fever Pathogen, Coxiella burnetii
Source: PLoS Pathog. 2015 May 15;11(5):e1004892. doi: 10.1371/journal.ppat.1004892 (PMC4433120; doi:10.1371/journal.ppat.1004892)
Supplement: S1 Text — Genes and primers used in polymerase chain reaction (PCR) assays to detect Coxiella and relatives and to control tick DNA quality. The same primers were used for the Coxiella and Rickettsiella typing, with the exception of the 16S rRNA gene for which different primers were designed for the two bacteria. Nested PCR amplifications (16S rRNA, 23S rRNA, GroEL, rpoB and dnaK) were performed as follows: the first PCR run with the external primers was performed in a 10μLvolume containing 20–50 ng of genomic DNA, 3 mM of each dNTP (Thermo Scientific), 8 mM of MgCl2 (Roche Diagnostics), 3 μM of each primer, 1 μL of 10× PCR buffer (Roche Diagnostics), and 0.5 U of Taq DNA polymerase (Roche Diagnostics). A 1-μL aliquot of the PCR product from the first reaction was then used as a template for the second round of amplification. The second PCR was performed in a total volume of 25 μL and contained 8 mM of each dNTP (Thermo Scientific), 10 mM of MgCl2 (ThermoScientific), 7.5 μM of each of the internal primers, 2.5 μL of 10×PCR buffer (Thermo Scientific), and 1.25 U of Taq DNA polymerase (Thermo Scientific). Non-nested PCR amplifications (CO1 and 18S rRNA) were performed following conditions similar to the first PCR run used in the nested PCR assays. All PCR amplifications were performed under the following conditions: initial denaturation at 93°C for 3 min, 35 cycles of denaturation (93°C, 30 s), annealing (Tm = 50–56°C, depending on primers, 30 s), extension (72°C, 1–2 min), and a final extension at 72°C for 5 min. Table B. List, biological features and GenBank accession numbers of the bacterial strains used as references in molecular and phylogenetic analyses.* reference strains of C. burnetii used for primer testing. Table C. List, sequence accession numbers and features of the 31 Coxiella contigs from the whole-genome shotgun sequencing (WGS) of the cattle tick Rhipicephalus microplus. Fig A. Map of the Coxiella burnetii genome (strain Nine Mile I RSA 493) showing the positio [file ppat.1004892.s001.docx]

**S1 Text**

The recent evolution of a maternally-inherited endosymbiont of ticks led to the emergence of the Q fever pathogen, *Coxiella burnetii*

**Table A.** Genes and primers used in polymerase chain reaction (PCR) assays to detect *Coxiella* and relatives and to control tick DNA quality. Nested PCR amplifications (*16S rRNA*, *23S rRNA*, *GroEL*, *rpoB* and *dnaK*) were performed as follows: the first PCR run with the external primers was performed in a 10µLvolume containing 20–50 ng of genomic DNA, 3 mM of each dNTP (Thermo Scientific), 8 mM of MgCl_2_ (Roche Diagnostics), 3 µM of each primer, 1 µL of 10× PCR buffer (Roche Diagnostics), and 0.5 U of Taq DNA polymerase (Roche Diagnostics). A 1-µL aliquot of the PCR product from the first reaction was then used as a template for the second round of amplification. The second PCR was performed in a total volume of 25 µL and contained 8 mM of each dNTP (Thermo Scientific), 10 mM of MgCl_2_ (ThermoScientific), 7.5 µM of each of the internal primers, 2.5 µL of 10×PCR buffer (Thermo Scientific), and 1.25 U of Taq DNA polymerase(Thermo Scientific). Non-nested PCR amplifications (CO1 and *18S rRNA*) were performed following conditions similar to the first PCR run used in the nested PCR assays. All PCR amplifications were performed under the following conditions: initial denaturation at 93◦C for 3 min, 35 cycles of denaturation (93°C, 30 s), annealing (Tm=50-56°C, depending on primers, 30 s), extension (72°C, 1-2 min), and a final extension at 72◦C for 5 min.

| Gene | | Hypothetical product | Primers (5'-3') | | Tm | Fragment size | Reference |
| --- | --- | --- | --- | --- | --- | --- | --- |
| Tick | | |  |  |  |  |  |
|  | 18S rRNA | Small ribosomal subunit | NSF4 - | CTGGTTGATYCTGCCAGT | 50°C | NSF4/NSR399: 404bp | [1,2] |
|  |  |  | NSR399 - | TCTCAGGCTCCYTCTCCGG |  |  |  |
|  | *C01* | cytochrome oxidase subunit I | Ron - | GGAGCYCCWGATATAGCTTTCCC | 50°C | Ron/Nancy: 488bp | This study; [3] |
|  |  |  | Nancy - | CCTGGTAAAATTAAAATATAAACTTC | | Alternative PCR: Ron/TCOIR: 463bp |  |
|  |  |  | TCOIF - | TTTTACCGCGATGAHTWTTYT |  | Alternative PCR: TCOIF/TCOIR: 237bp |  |
|  |  |  | TCOIR - | WGGRTGRCCAAARAATCAAAATA |  | Alternative PCR: TCOIF/Nancy: 262bp |  |
| *Coxiella* | |  |  |  |  |  |  |
|  | 16S rRNA | Small ribosomal subunit | Cox16SF1 - | CGTAGGAATCTACCTTRTAGWGG | 52-56°C | 1st PCR run: Cox16SF1/Cox16SR2: 1321-1429bp (for *Coxiella* only) | This study; [4] |
|  |  |  | Cox16SF2 - | TGAGAACTAGCTGTTGGRRAGT |  | Alternative 1st PCR run: 16S_07F/Cox16SR2: 1434-1542bp (for *Coxiella* only) |  |
|  |  |  | 16S_07F - | AGAGTTTGATYMTGGCTCAG |  | 2nd PCR run (fragment 1): Cox16SF1/Cox16SR1: 719-826bp (for *Coxiella* only) |  |
|  |  |  | Cox16SR1 - | ACTYYCCAACAGCTAGTTCTCA |  | Alternative 2nd PCR run (fragment 1): 16S_07F/Cox16SR1: 832-939bp (for *Coxiella* only) |  |
|  |  |  | Cox16SR2 - | GCCTACCCGCTTCTGGTACAATT |  | 2nd PCR run (fragment 2): Cox16SF2/Cox16SR2: 624-627bp (for *Coxiella* only) |  |
|  |  |  | Rla16SF1 - | CAGTAAARRTTTCGGYCTTTAYGGG |  | 1st PCR run: Rla16SF1/Rla16SR2: 1224-1227bp (for *Rickettsiella* only) |  |
|  |  |  | Rla16SF2 - | CGTGTAGGTGGTTGACTAGGTTTG |  | 2nd PCR run (fragment 1): Rla16SF1/Rla16SR1: 532-534bp (for *Rickettsiella* only) |  |
|  |  |  | Rla16SR1 - | CAAACCTAGTCAACCACCTACACG |  | 2nd PCR run (fragment 2): Rla16SF2/Rla16SR2: 716-718bp (for *Rickettsiella* only) |  |
|  |  |  | Rla16SR2 - | GGATTGGCTCCCCCTCGCGGGTTGG |  |  |  |
|  | 23S rRNA | Large ribosomal subunit | Cox23SF1 - | GCCTGCGAWAAGCTTCGGGGAG | 56°C | 1st PCR run: Cox23SF1/Cox23SR2: 694-1188bp | This study |
|  |  |  | Cox23SF2 - | GATCCGGAGATWTCYGAATGGGG |  | 2nd PCR run: Cox23SF2/Cox23SR1: 583-867bp |  |
|  |  |  | Cox23SR1 - | TCGYTCGGTTTCGGGTCKACTC |  | Alternative 2nd PCR run: Cox23SF1/Cox23SR2: 601-884bp |  |
|  |  |  | Cox23SR2 - | CTCCTAKCCACASCTCATCCCC |  |  |  |
|  | *GroEL* | Chaperone protein GROEL | CoxGrF1 - | TTTGAAAAYATGGGCGCKCAAATGGT | 56°C | 1st PCR run: CoxGrF1/CoxGrR2: 655bp | [4] |
|  |  |  | CoxGrR2 - | CGRTCRCCAAARCCAGGTGC |  | 2nd PCR run: CoxGrF2/CoxGrR1: 619bp |  |
|  |  |  | CoxGrF2 - | GAAGTGGCTTCGCRTACWTCAGACG |  |  |  |
|  |  |  | CoxGrFR1 - | CCAAARCCAGGTGCTTTYAC |  |  |  |
|  | *rpoB* | DNA-directed RNA polymerase beta chain | CoxrpoBF2 - | GGGCGNCAYGGWAAYAAAGGSGT | 56°C | 1st PCR run: CoxrpoBF2/CoxrpoBR1: 607-610bp | This study |
|  |  |  | CoxrpoBR1 - | CACCRAAHCGTTGACCRCCAAATTG |  | 2nd PCR run: CoxrpoBF3/CoxrpoBR3: 539-542bp |  |
|  |  |  | CoxrpoBF3 - | TCGAAGAYATGCCYTATTTAGAAG |  |  |  |
|  |  |  | CoxrpoBR3 - | AGCTTTMCCACCSARGGGTTGCTG |  |  |  |
|  | *dnaK* | Chaperone protein DNAK | CoxdnaKF1 - | CGTCARGCRACGAARGATGCA | 54°C | 1st PCR run: CoxdnaKF1/CoxdnaKR: 777bp | This study |
|  |  |  | CoxdnaKF2 - | GAAGTGGATGGCGARCAYCAATT |  | Alternative 1st PCR run: CoxdnaKF3/CoxdnaKR: 636bp |  |
|  |  |  | CoxdnaKF3 - | GGTACKTTYGATATTTCCATC |  | 2nd PCR run: CoxdnaKF2/CoxdnaKR: 603bp |  |
|  |  |  | CoxdnaKR - | CGTCATGAYKCCGCCYAAGG |  | Alternative 2nd PCR run: CoxdnaKF2/CoxdnaKR3: 512bp |  |
|  |  |  | CoxdnaKR3 - | CTTGAATAGCYGCACCAATAGC |  |  |  |

References

1. Hendriks L, De Baere R, Van de Peer Y, Neefs J, Goris A, De Wachter R. The evolutionary position of the rhodophyta *Porphyra umbilicalis* and the basidiomycete *Leucosporidium scottii* among other eukaryotes as deduced from complete sequences of small ribosomal subunit RNA. J Mol Evol. 1991;32: 167-177.

2. Hendriks L, Goris A, De Bruyn K, De Wachter R. The nucleotide sequence of the small ribosomal subunit RNA of the yeast *Torulaspora delbrueckii*. Nucleic Acids Res 1990;18: 4611.

3. Simon C, Frati F, Beckenbach A, Crespi B, Liu H, Flook P. Evolution, weighting, and phylogenetic utility of mitochondrial gene sequences and a compilation of conserved polymerase chain reaction primers. Ann Entomol Soc Am. 1994;87: 651–701.

4. Duron O, Jourdain E, McCoy KD. Diversity and global distribution of the *Coxiella* intracellular bacterium in seabird ticks. Ticks Tick Borne Dis. 2014;5: 557-563.

**Table B.** List, biological features and GenBank accession numbers of the bacterial strains used as references in molecular and phylogenetic analyses.* reference strains of *C. burnetii* used for primer testing.

| Bacteria | Isolation source | Biological feature | GenBank accession number |
| --- | --- | --- | --- |
| *Coxiella burnetii* (strain Nine Mile I / RSA 493)* | Tick, USA, 1935 | Human acute Q Fever (high virulence) | AE016828 |
| *Coxiella burnetii* (strain Henzerling / RSA 331) | Human, Italy, 1945 | Human acute Q Fever | CP000890 |
| *Coxiella burnetii* (strain Dugway / 5J108‑111) | Rodent, USA, 1958 | Low virulence in guinea pigs; not determined in humans | CP000733 |
| *Coxiella burnetii* (strain CbuK / Q154) | Human, USA, 1976 | Human chronic Q Fever (endocarditis) | CP001020 |
| *Coxiella burnetii* (strain CbuG / Q212) | Human, Canada, 1982 | Human chronic Q Fever (endocarditis) | CP001019 |
| *Coxiella burnetii* (strain Guyana / Cb175) | Guyana | Unknown | CAVF010000000 |
| *Coxiella burnetii* (strain Cb109) | Human, Germany | Human chronic Q Fever (endocarditis) | AKYP00000000 |
| *Coxiella burnetii* (strain Z3055) | Goat, Germany | Goat abortion (epidemic form) | CAUE01000000 |
| *Coxiella burnetii* (strain Cb_B1)* | Cattle, Europe | Cattle abortion | CCAH010000000 |
| *Coxiella burnetii* (strain Cb_B18) | Cattle, Europe | Asymptomatic | CCAI00000000 |
| *Coxiella burnetii* (strain Cb_C2)* | Goat, Europe | Asymptomatic | CCAJ000000000 |
| *Coxiella burnetii* (strain Cb_O184)* | Sheep, Europe | Sheep abortion | CCAK000000000 |
| *Coxiella burnetii* (strain EV-Cb_BK10) | Cattle, Europe | Asymptomatic | CCAL000000000 |
| *Coxiella burnetii* (strain EV-Cb_C13) | Goat, Germany | Goat abortion | CCAM000000000 |
| *Coxiella burnetii* (strain Cb185) | Human | Letal fœtal malformation | CBTH010000000 |
| *Rickettsiella grylli* | Woodlice | Unknown | AAQJ02000000 |
| *Legionella pneumophila* (strain Paris) | Human | Legionnaires' disease | CR628336 |
| *Legionella longbeachae* (strain NSW150) | Human | Legionnaires' disease | NC013861 |
| *Escherichia coli* (strain MG1655) | Human | Human intestinal microflora | NC000913 |
| *Salmonella enterica* (serovar Typhimurium) | Human | Human gastroenteritis | NC003197 |

**Table C.** List, sequence accession numbers and features of the 31 *Coxiella* contigs from the whole-genome shotgun sequencing (WGS) of the cattle tick *Rhipicephalus microplus*.

| *Coxiella* contigs from the *Rhipicephalus microplus* WGS (GenBank accession number) | | Size (bp) | Gene | Putative gene product | Matching locus tag in the *Coxiella burnetii* genome (str. Nine Mile I RSA 493, GenBank accession number AE016828) | Percentage identity with *Coxiella burnetii* |
| --- | --- | --- | --- | --- | --- | --- |
| 1 | ADMZ02082630 | 2349 | *rpoB* | DNA-directed RNA polymerase beta chain | CBU_0231 | 78 |
| 2 | ADMZ02065298 | 1204 | *nusA* | Transcription termination factor | CBU_1433 | 79 |
| 3 | ADMZ02074966 | 1474 | *acnA* | Aconitate hydratase | CBU_1720 | 75 |
|  |  |  | *GroES* | 10 kDa chaperonin GROES | CBU_1719 | 80 |
|  |  |  | *GroEL* | 60 kDa chaperonin GROEL | CBU_1718 | 81 |
| 4 | ADMZ02052590 | 1022 | *rpoC* | DNA-directedRNA polymerase beta' subunit | CBU_0232 | 76 |
| 5 | ADMZ02027523 | 832 | *23S rRNA* | Large Subunit Ribosomal RNA | CBU_23S | 92 |
| 6 | ADMZ02020628 | 795 | *fusA* | Elongation factor G | CBU_0235 | 79 |
| 7 | ADMZ02057878 | 1086 | *prsA* | Ribose-phosphate pyrophosphokinase | CBU_1830 | 75 |
|  |  |  | *tRNA-Gln* | tRNA-Gln | CBU_tRNA-Gln-1 | 100 |
| 8 | ADMZ02063767 | 1643 | *gcvPB* | Glycine dehydrogenase subunit 2 (decarboxylating) | CBU_1713 | 77 |
| 9 | ADMZ02060773 | 1127 | *ssb* | Single-strand DNA binding protein | CBU_0271 | 84 |
|  |  |  | *yajR* | Transporter, MFS superfamily | CBU_0272 | 80 |
| 10 | ADMZ02056534 | 1069 | *rpmF* | LSU ribosomal protein L32P | CBU_0491 | 80 |
|  |  |  | *plsX* | Fatty acid/phospholipid synthesis protein | CBU_0492 | 77 |
| 11 | ADMZ02077074 | 1579 | *xth* | Exodeoxyribonuclease III | CBU_0297 | 77 |
|  |  |  | *pyrE* | Orotate phosphoribosyltransferase | CBU_0296 | 86 |
| 12 | ADMZ02175154 | 710 | *dnaZX* | DNA polymerase III subunit gamma/tau | CBU_0659 | 76 |
|  |  |  | unnamed gene | Hypothetical protein | CBU_0660 | 92 |
| 13 | ADMZ02051438 | 1011 | *fabG* | 3-oxoacyl-reductase | CBU_0495 | 83 |
|  |  |  | *acpP* | Acyl carrier protein | CBU_0496 | 86 |
|  |  |  | *fabF* | 3-oxoacyl-synthase | CBU_0497 | 74 |
| 14 | ADMZ02081523 | 2035 | unnamed gene | NAD-specific glutamate dehydrogenase | CBU_1226 | 76 |
| 15 | ADMZ02168346 | 687 | *tRNA-Cys* | tRNA-Cys | CBU_tRNA-Cys-1 | 94 |
|  |  |  | unnamed gene | Nucleotidyltransferase | CBU_1021 | 76 |
| 16 | ADMZ02093396 | 514 | *yibO* | Phosphoglycerate mutase | CBU_1536 | 73 |
| 17 | ADMZ02031963 | 857 | *rpiA* | Ribose 5-phosphate isomerase | CBU_0026 | 70 |
| 18 | ADMZ02042842 | 932 | *tRNA-Leu* | tRNA-Leu | CBU_tRNA-Leu-1 | 95 |
| 19 | ADMZ02074095 | 1439 | *tRNA-Pro* | tRNA-Pro | CBU_tRNA-Pro-2 | 100 |
|  |  |  | *tRNA-Arg* | tRNA-Arg | CBU_tRNA-Arg-1 | 96 |
|  |  |  | *dapD* | 2,3,4,5-tetrahydropyridine-2-carboxylate N-succinyltransferase | CBU_0667 | 77 |
| 20 | ADMZ02008781 | 743 | *tRNA-Trp* | tRNA-Trp | CBU_tRNA-Trp-1 | 97 |
|  |  |  | *secE* | Protein translocase subunit | CBU_0224 | 76 |
|  |  |  | *nusG* | Transcription termination/antitermination factor NusG | CBU_0225 | 74 |
| 21 | ADMZ02063385 | 1169 | *tRNA-Glu* | tRNA-Glu | CBU_tRNA-Glu-1 | 99 |
|  |  |  | *sixA* | Phosphohistidine phosphatase SixA homolog | CBU_0099 | 68 |
| 22 | ADMZ02038422 | 900 | *tRNA-Met* | tRNA-Met | CBU_tRNA-Met-1 | 99 |
| 23 | ADMZ02031754 | 856 | *tRNA-Arg* | tRNA-Arg | CBU_tRNA-Arg-2 | 99 |
| 24 | ADMZ02079929 | 1799 | *tRNA-Ser* | tRNA-Ser | CBU_tRNA-Ser-2 | 98 |
|  |  |  | *csrA-2* | Carbon storage regulator | CBU_1050 | 87 |
|  |  |  | unnamed gene | Aspartokinase | CBU_1051 | 73 |
| 25 | ADMZ02038274 | 899 | *accA* | Acetyl-coenzyme A carboxylase carboxyl transferase subunit alpha | CBU_1510 | 77 |
|  |  |  | unnamed gene | ZIP family zinc transporter | CBU_1511 | 74 |
| 26 | ADMZ02017471 | 780 | *dnaA* | Chromosomal replication initiator protein | CBU_0001 | 82 |
| 27 | ADMZ02034469 | 873 | *leuS* | Leucyl-tRNA synthetase | CBU_0559 | 76 |
| 28 | ADMZ02156645 | 651 | non coding sequence | none | none (between CBU_1308 and CBU_1308a) | 78 |
| 29 | ADMZ02068099 | 1262 | unnamed gene | GTP-binding protein, probable translation factor | CBU_1842 | 70 |
|  |  |  | *tRNA-Met* | tRNA-Met | CBU_tRNA-Met-3 | 91 |
| 30 | ADMZ02081020 | 1946 | unnamed gene | Peptidoglycan-specific endopeptidase, M23 family | CBU_1670 | 68 |
|  |  |  | *surE* | Acid phosphatase | CBU_1671 | 74 |
|  |  |  | *ftsB* | Cell division protein | CBU_1673 | 86 |
| 31 | ADMZ02009803 | 747 | non coding sequence | none | none (between CBU_1552 and CBU_1552a) | 93 |

**Figure legends**

**Fig A.** Map of the *Coxiella burnetii* genome (strain Nine Mile I RSA 493) showing the position of the genetic markers (in blue) used in this study. The arrows indicate the position along the chromosome of the five housekeeping genes (16S rRNA, 23S rRNA, *GroEL*, *rpoB* and *dnaK*) used in the multi-locus typing of tick-borne *Coxiella* infections. The numbered boxes (1-31) indicate the position of the 31 *Coxiella* contigs (listed in Table B in S1 Text) detected from the whole genome sequencing of the hard tick *Rhipicephalus microplus*.

**Fig B.** *Coxiella* and *Rickettsiella* phylogeny constructed using maximum-likelihood (ML) estimations based on 16S rRNA, 23S rRNA, *GroEL*, *rpoB* and *dnaK* concatenated sequences (3009bp), including 71 Coxiella-like strains of ticks, 15 *C. burnetii* reference strains and outgroups. The four *Coxiella* clades are labeled A to D. Each number corresponds to one tick species as detailed in Table 1. Blue, *Coxiella*-line organisms; red, *C. burnetii*; green, *Rickettsiella*; black, other bacteria. All multi-locus typing of tick-borne *Coxiella* and *Rickettsiella* of ticks are new sequences from this study. Branch numbers indicate percentage bootstrap support for major branches (1000 replicates; only bootstrap values >90% are shown).

**Fig C.** Inset of *Coxiella* network from Fig 2 with focus on the A clade (*Coxiella* of soft ticks and *C. burnetii*). Each number corresponds to one tick species as detailed in Table 1. Blue, *Coxiella*-line organisms; red, *C. burnetii.* The scale bar is in units of substitution/site.

**Fig A**

**Fig B**

**Fig C**
